# Detection and characterization of ESBL-producing *Enterobacteriaceae* from the gut of subsistence farmers, their livestock, and the surrounding environment in rural Nepal

**DOI:** 10.1038/s41598-021-81315-3

**Published:** 2021-01-22

**Authors:** Supram Hosuru Subramanya, Indira Bairy, Yang Metok, Bharat Prasad Baral, Dipendra Gautam, Niranjan Nayak

**Affiliations:** 1 Department of Medical Microbiology, Manipal College of Medical Sciences, Pokhara, Nepal; 2 Melaka Manipal Medical College, Manipal Academy of Higher Education, Manipal, India

**Keywords:** Antimicrobial resistance, Microbiology, Medical research

## Abstract

The increasing trend of gut colonization by extended-spectrum β-lactamase (ESBL) producing Enterobacterales has been observed in conventional farm animals and their owners. Still, such colonization among domesticated organically fed livestock has not been well studied. This study aimed to determine the gut colonization rate of ESBL-producing *Enterobacteriaceae* and carbapenemase-producing *Enterobacteriaceae* (CPE) among rural subsistence farming communities of the Kaski district in Nepal. Rectal swabs collected by systematic random sampling from 128 households of subsistence farming communities were screened for ESBL-producing *Enterobacteriaceae* and CPE by phenotypic and molecular methods. A total of 357 (57%) ESBL-producing *Enterobacteriaceae* isolates were obtained from 626 specimens, which included 97 ESBL-producing *Enterobacteriaceae* (75.8%) from 128 adult humans, 101 (79.5%) from 127 of their children, 51 (47.7%) from 107 cattle, 26 (51%) from 51 goats, 30 (34.9%) from 86 poultry and 52 (42%) from 127 environmental samples. No CPE was isolated from any of the samples. *bla*_CTX-M-15_ was the most predominant gene found in animal (86.8%) and human (80.5%) isolates. Out of 308 *Escherichia coli* isolates, 16 human and two poultry isolates were positive for ST131 and were of clade C. Among non-cephalosporin antibiotics, the resistance rates were observed slightly higher in tetracycline and ciprofloxacin among all study subjects. This is the first one-health study in Nepal, demonstrating the high rate of CTX-M-15 type ESBL-producing *Enterobacteriaceae* among gut flora of subsistence-based farming communities. Gut colonization by *E. coli* ST131 clade C among healthy farmers and poultry birds is a consequential public health concern.

## Introduction

Antimicrobial resistance (AMR) has become a severe threat worldwide due to the global emergence of new resistance mechanisms and limited drugs available for treatment^1^. In low-and middle-income countries (LMICs), the extent and the impact of antibiotic resistance tend to be even more significant than in developed countries^2^. The rates of multi-drug resistance in human and animal pathogens are steadily rising around the globe. These resistance trends are also extending to the commensal microbial flora of human, animal, and environmental origin, which is considered a severe public health threat^3^.

Antibiotics consumed by humans and animals intensely alter their normal microbial flora and select out such strains rendering increased resistance to these molecules^4^. Irrational use of various antimicrobial drugs, including those used in human medication and animal husbandry, augment the development of resistance to a wide range of antimicrobial agents. It is believed that farm animals may act as reservoirs for the spread of drug resistance genes. A recent study points to the environment as a significant component for the emergence and dissemination of multidrug-resistant (MDR) bacteria. However, there is still a lack of detailed understanding of evolutionary and ecological processes in the emergence of resistance genes and environmental dispersal barriers^5^.

In the early 1980s, the first strains of ESBL-producing *Enterobacteriaceae* were reported immediately after broad-spectrum cephalosporins were approved for clinical usage^6,7^. However, the emergence of such resistant traits was restricted to healthcare-associated isolates confined within the clinical settings^8^. Gradually, this trend shifted towards community-associated clinical isolates, and now ESBL-producing *Enterobacteriaceae* have been disseminating to the community inhabitants, including the gut flora of healthy humans and animals. Carbapenem-resistant *Enterobacteriaceae* (CRE) are common in hospital settings and are infrequently reported at the community level^9^. The *Enterobacteriaceae* members, particularly *Escherichia coli* and *Klebsiella* spp., are human and animal gut flora; these florae may serve as quiescent reservoirs of transmissible antimicrobial resistance to the pathogens. Acquisition of ESBL/carbapenemase genes among these gut flora plays a central role in the spread of MDR bacteria among humans, animals, and the environment via the food chain. The gut colonization with ESBL-producing *Enterobacteriaceae*/CRE may serve as a vivacious source for horizontal transmission and endogenous infections^10^. Therefore, such resistant gut flora can no longer be considered as innocent bystanders.

Currently, *Enterobacteriaceae* members' harboring cefotaxime (CTX)-M enzymes are the most dominant strains associated with global ESBLs. *E. coli*, with CTX-M-15, is the most common CTX-M type ESBLs, followed by CTX-M-14, which are highly prevalent in Asia^7^. Mobile genetic elements like insertion elements, integrons, and transposons have played a significant role in capturing and mobilizing CTX-M encoding genes onto different types of plasmids, which assist the spread of ESBLs to a wide variety of hosts^9^. The population structure of ESBL producing *E. coli* is influenced by ST131 clones (typical extra-intestinal pathogenic *E. coli* clone), which are the quintessential examples of successful high-risk clones disseminating globally in human clinical isolates^9^.

Combating the increasing trends in the prevalence of ESBL-producing *Enterobacteriaceae* and CRE infections in humans requires understanding reservoirs and sources for human acquisition. *E. coli* present in humans and animals' gut is considered an indicator, which provides hints on the emergence and dissemination of drug resistance. The frequency of resistance in commensal *E. coli* is considered a useful marker for the selective pressure applied by antibiotic use and the future resistance predicted in pathogens^11^. LMICs have a high AMR burden, and optimal interventions are hampered by a lack of surveillance^12^. The chances of transmission (bacteria and/or resistance determinants) between humans and animals can be hypothesized as a consequence of close association between owners/caretakers and their livestock in LMICs. The one-health perspective studies on gut flora are imperative in addressing the flow of antimicrobial resistomes in different ecosystems and their interface. The one-health AMR surveillance study is disparagingly essential in low-income countries like Nepal, where most people live in rural areas with low socioeconomic status and rely primarily on agriculture and farming as a source of income^13^.

In conventional farming, various antimicrobial compounds have been used as therapeutic, metaphylaxis, and prophylactic agents to help livestock grow faster and prevent infection and death^14^. The majority of previous studies^15^ have documented an increased prevalence of MDR bacterial colonization in conventional farm animals and their owners around the globe. Still, such carriage rates among organically fed livestock have not been extensively studied. Therefore, inclusions of backyard/non-conventional farm animals and poultry birds in surveillance for ESBL and CRE are essential to trace the reservoirs and limit their spread. In Nepal, one-health AMR surveillance studies are sparse, and the proportion of gut colonization by ESBL-producing *Enterobacteriaceae* and Carbapenemase-producing *Enterobacteriaceae* (CPE) in humans and animals is not well documented. Therefore, we conducted a cross-sectional study under a one-health approach to determine the occurrence of ESBL-producing *Enterobacteriaceae* and CRE among rural subsistence farming communities of the Kaski district in Nepal.

## Results

Out of a total of 626 specimens collected from 128 subsistence farming communities, 366 (58.5%, 95% CI (54.5%, 62.4%)) samples yielded presumptive ESBL-positive organisms. No carbapenem-resistant *Enterobacterale* grew on selective plates*.* Of these 366 ESBL-positive specimens, 97 (75.8%) were obtained from 128 adults, 101 (79.5%) from 127 of their children, 51 (47.7%) from 107 cattle, 26 (50.9%) from 51 goats, 30 (34.9%) from 86 poultry and 61 (48%) from 127 environmental sources (Table 1). Twenty-eight samples yielded two different types of presumptive ESBL-producing bacteria (*E. coli* or *Klebsiella* spp. or *Enterobacter* spp. or *Citrobacter* spp.), thus accounting for 394 ESBL-producing *Enterobacteriaceae* isolates from 366 specimens. All 394 isolates were phenotypically confirmed as ESBL producers. None of the isolates was carbapenemase-producer by phenotypic tests. The number (percentage) of ESBL-producing *Enterobacteriaceae* isolated from humans, animals, and the environment is shown in Table 1. Certainly, *E. coli* were found to be the predominant flora, followed by *Klebsiella* spp., *Enterobacter* spp., and *Citrobacter* spp. in all groups of study subjects (Table 2).

**Table 1:** Percentage of presumptive ESBL-positive specimens and number of ESBL-producing *Enterobacteriaceae* strains isolated from 626 samples of 128 subsistence farming communities.

Sl. No	Specimens collected from subsistence farming communities (n = 128)	ESBL-positive specimens (% (CI))	ESBL-producing *Enterobacteriaceae* strains isolated
1	Total Specimen: 626	366 (58.5% (54.5%, 62.4%))	394
2	Adults: 128	97 (75.8% (67.4%, 82.9%))	105
3	Children: 127	101 (79.5% (71.5%, 86.2%))	111
4	**Animals: 244** Buffalo/ Cow: 107 Goat: 51 Poultry: 86	107 (43.9% (37.5%, 50.3%)) 51 (47.7% (37.9%, 57.5%)) 26 (51% (36.6%, 65.2%)) 30 (34.9% (24.9%, 45.9%))	113 52 26 35
5	Environment: 127	61(48% (39.1%, 57.1%))	65

**Table 2:** Number (%) of ESBL-producing *Enterobacteriaceae* isolated from human, animal, and environmental specimen.

	Adult	Child	Buffalo/cow	Goat	Poultry	Environment
Total ESBL-producing *Enterobacteriaceae* isolates	**105**	**111**	**52**	**26**	**35**	**65**
*E. coli* (334)	84(80%)	90(81%)	46(88.5%)	26(100%)	28(80%)	60(92.3%)
*Klebsiella* spp. (47)	16(15.2%)	14 (12.6%)	6(11.5%)	0(0%)	6(17.1%)	5(7.7%)
*Enterobacter* spp. (9)	3(2.9%)	5(4.5%)	0(0%)	0(0%)	1(2.86%)	0(0%)
*Citrobacter* spp. (4)	2(1.9%)	2(1.8%)	0(0%)	0(0%)	0(0%)	0(0%)

### Antibiotic susceptibility testing

**Figure 1:**
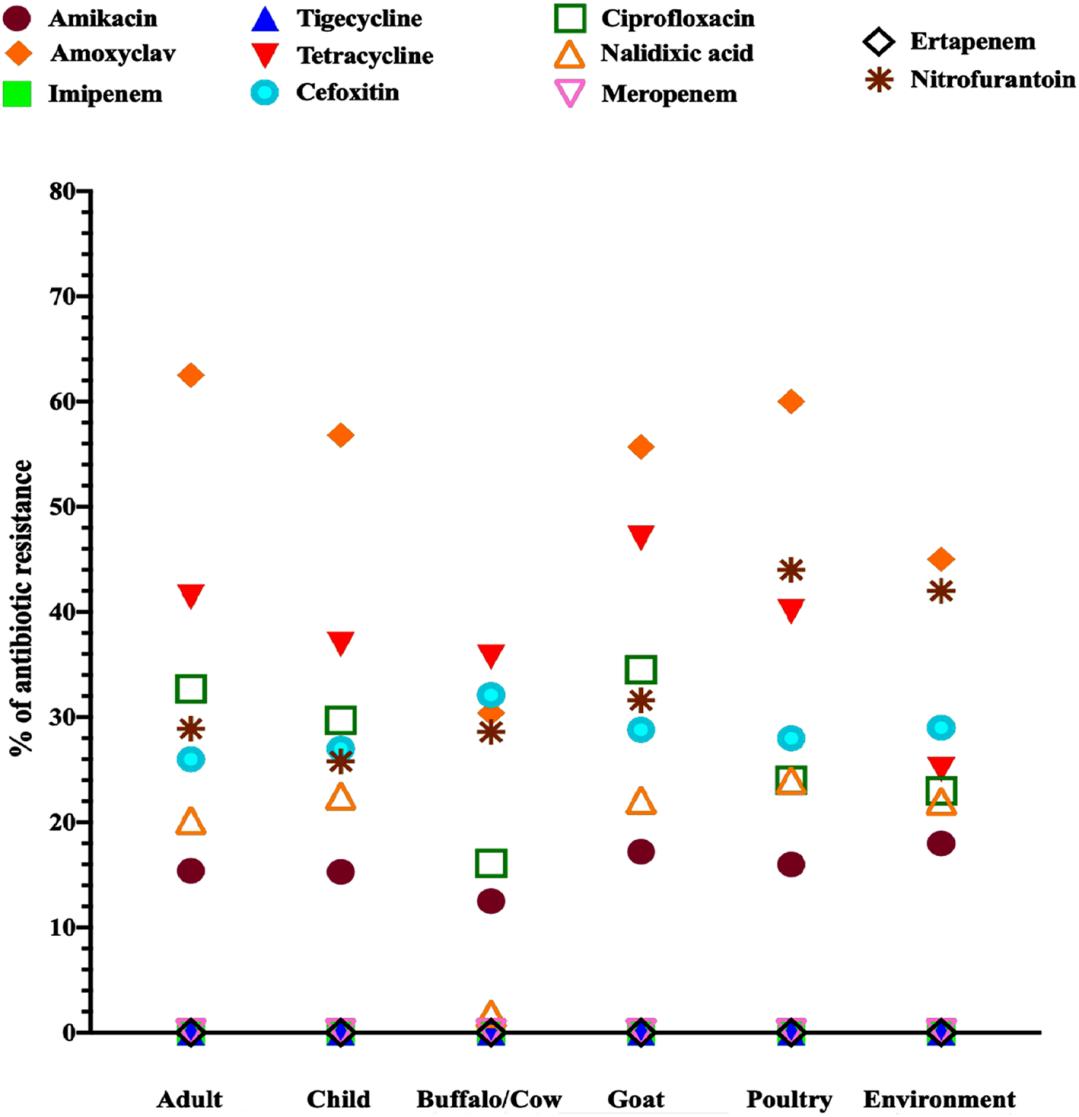
Antibiotic resistance pattern of ESBL-producing *Enterobacteriaceae* strains isolated from different groups of the community.

All the 394 ESBL-producing *Enterobacteriaceae* isolates were tested for susceptibility to various antibiotics. The antibiotic resistance profile of ESBL-producing *Enterobacteriaceae* isolates of each group is presented in Fig. 1, supplementary file 7: Table S4. Besides cephalosporin antibiotics, the highest rate of resistance was found against amoxicillin/clavulanic acid (63.1% of adult, 28.2% of cattle ESBL-producing *Enterobacteriaceae* isolates) followed by tetracycline (47% of the goat isolates, 25% of the environmental isolates) and ciprofloxacin (34.5% of the goat isolates, 14.9% of cattle isolates). Among animals, the poultry isolates showed the highest resistance to tested antibiotics, and the resistance rate of these poultry isolates towards nitrofurantoin was found to be maximum. All the ESBL isolates were susceptible to tigecycline, imipenem, meropenem, and ertapenem (Figs. 1, 4).

### Multi-drug resistance pattern

**Figure 2:**
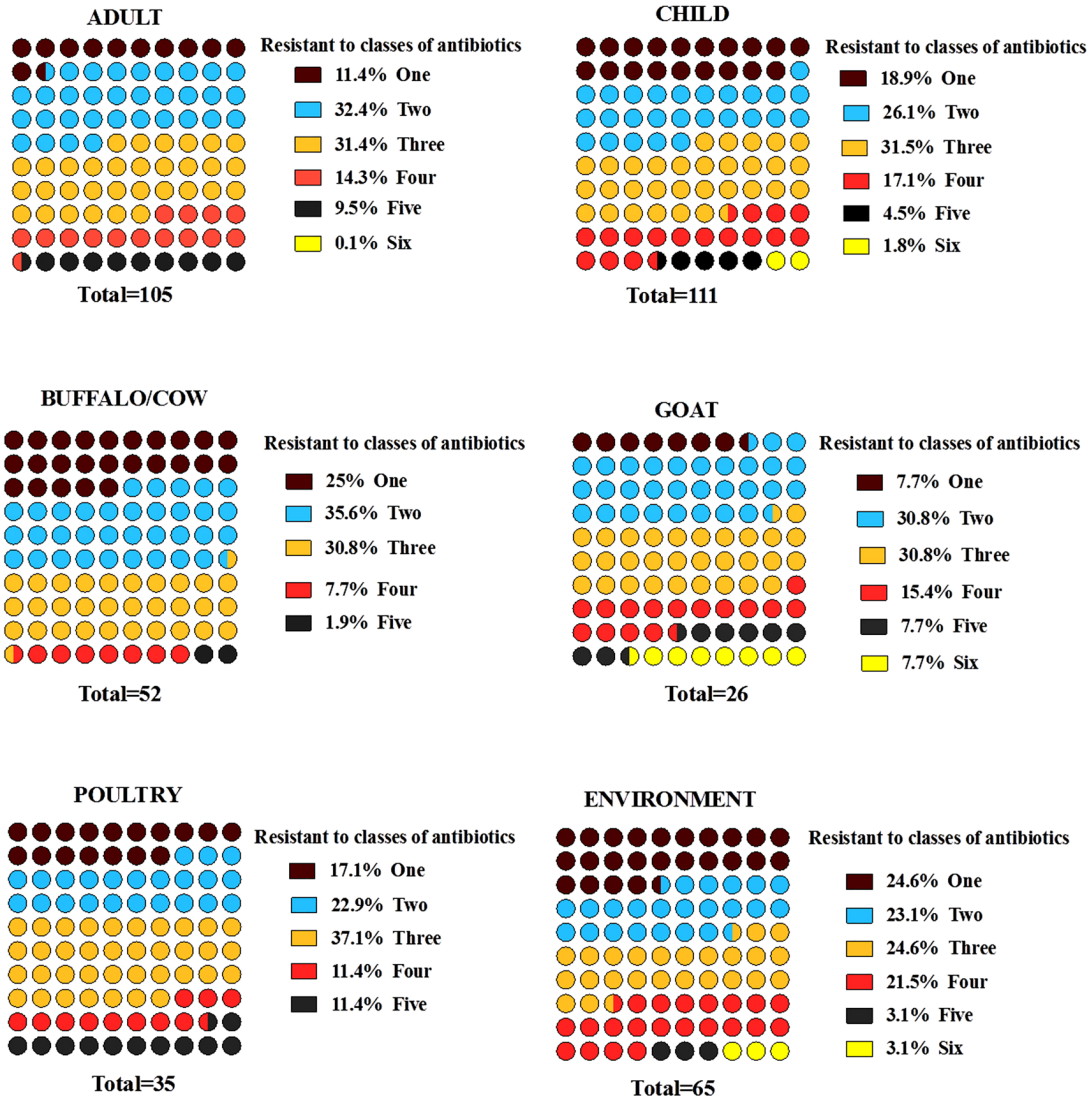
Multidrug resistance pattern of ESBL-producing *Enterobacteriaceae* strains isolated from 128 subsistence farming communities (humans, livestock, and their surrounding environment).

As shown in Fig. 2 and supplementary file 1: Fig. S1, the highest multi-drug resistance (non-susceptibility to at least one drug in three or more antibiotic categories) level was observed among the isolates of goat (61.5%) followed by poultry (60%). Adult (56.2%), child (55%), and environmental (52.3%) isolates had shown an almost similar level of multi-drug resistance. The lowest MDR percentage was found among the strains of cattle (39.4%). However, 7.7% of the goat, 3.1% of the environment, and 1.8% of child ESBL-producing *Enterobacteriaceae* isolates showed resistance to six antibiotics classes.

### Diversity among ESBL genes

**Figure 3:**
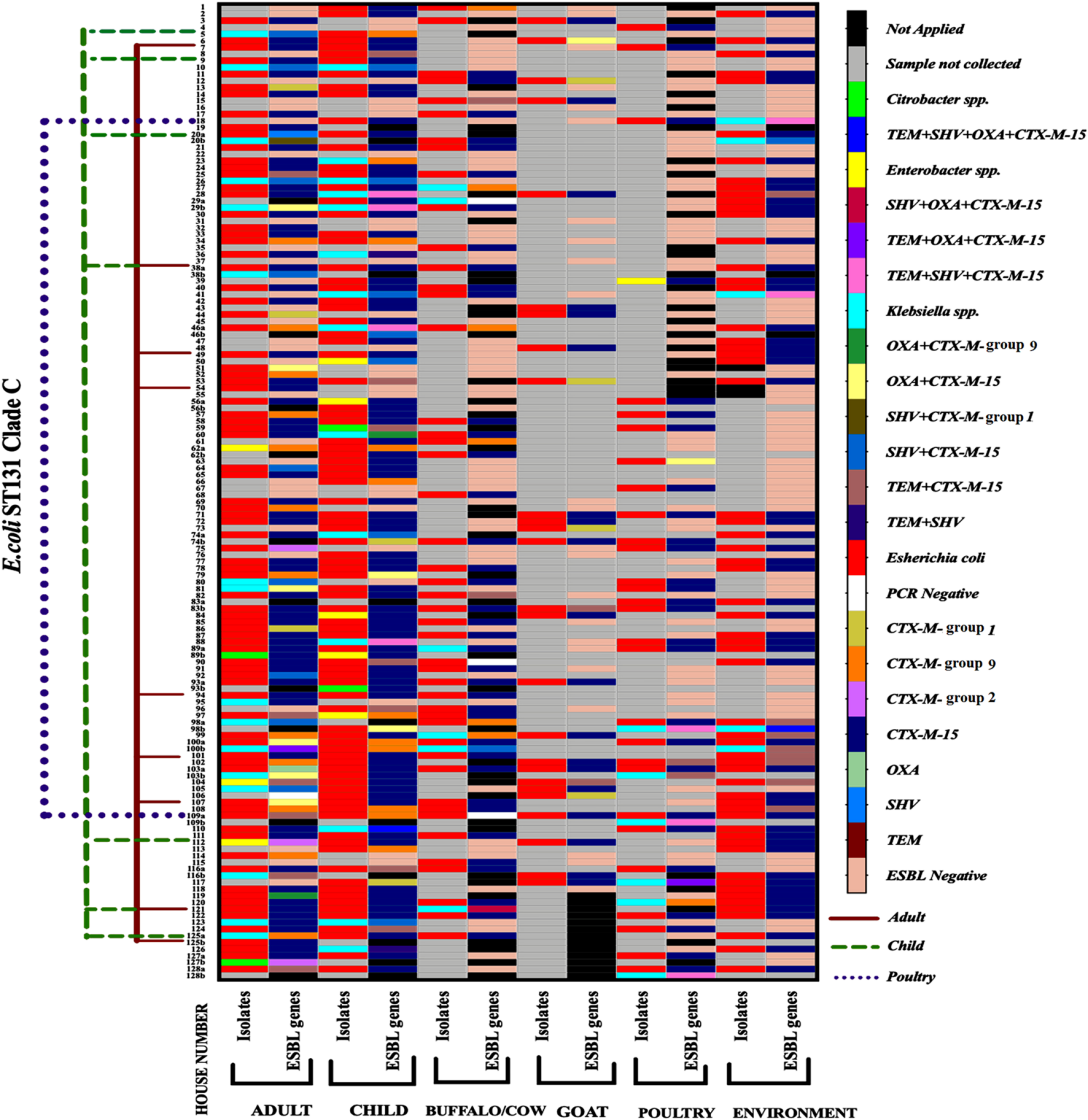
ESBL related genes and *E. coli* ST131 high-risk clones identified in 394 ESBL-producing *Enterobacteriaceae* strains isolated among six groups of subsistence farming communities. **TEM**: TEM variants including *bla*_TEM-1 and TEM-2_; **SHV**: SHV variants including *bla*_SHV-1_; **OXA-1 like**: *bla*_OXA-1,_ *bla*_OXA-4,_ and *bla*_OXA-30_; **CTX-M group 1**: variants of CTX-M group 1 (includes *bla*_CTX-M-1/3,_ excluding *bla*_CTX-M-15);_ **CTX-M group 2**: variants of CTX-M group 2 (includes *bla*_CTX-M-2_); CTX-M group 9: variants of CTX-M group 9 (includes *bla*_CTX-M-9/14_).

All 394 phenotypically ESBL-positive isolates were subjected to genotypic characterization via multiplex PCR assay. Among *bla*_CTX-M_ type ESBLs, 80% (84/105) of adult ESBL-producing *Enterobacteriaceae* isolates, 86.5% (96/111) of the child, 84.6% (44/52) of cattle, 100% (26/26) of goat, 97.1% (34/35) of poultry, and 100% (65/65) of environmental ESBL-producing *Enterobacteriaceae* isolates belonged to phylogenetic group 1 (variants of CTX-M group-1 including CTX-M-1, CTX-M-3, and CTX-M-15). A total of 12.3% (13/105) of adults and 10.8% (12/111) of children ESBL-producing *Enterobacteriaceae* isolates, 11.5% of cattle, and 2.9% of poultry possessed CTX-M phylogenetic group-9 (variants of CTX-M group-9 including CTX-M-9 and CTX-M-14). However, isolates from the goat and environment were negative for CTX-M group-9. CTX-M phylogenetic group-2 (variants of CTX-M group-2 including CTX-M-2) was found only in three (2.8%) isolates of the adult population. Further characterization of phylogroup-1 positive ESBL-producing *Enterobacteriaceae* isolates revealed that *bla*_CTX-M-15_ was the most predominant gene found in the environmental (100%; 65/65), animal (cattle: 100%; 44/44, goat: 84.6%; 22/26, poultry: 100%; 34/34), and human (adult: 80/84; 95.2% and child: 94/96; 97.9%) isolates. TEM was found in 10.2% of human isolates, 8.8% of animals, and 16.9% of environmental isolates. Only 9.5% and 8.6% of adult isolates, 11.7% and 3.6% of child isolates, 1.8% (2/113) and 2.7% (3/113) of animal isolates, and 6.2% (4/65) and 1.5% (1/65) of environmental isolates carried *bla*_SHV_ and *bla*_OXA-1_ respectively (Fig. 3). Most of the isolates possessing TEM, SHV, and OXA-1 co-harbored the CTX-M group. Compared to environmental (18.5%) and animal (10.6%) isolates, human isolates (20.8%) co-harbored higher level of multiple beta-lactamase genes. The most common multiple genes in animal and environmental isolates were *bla*_CTX-M-15_ + *bla*_TEM_ (12.3%). However, *bla*_SHV_ + *bla*_CTXM-15_ was the most prevalent multiple gene combinations in human isolates (6.5%) (supplementary file 2: Tables S1, S2).

### *E. coli* ST131 high-risk clones

**Figure 4:**
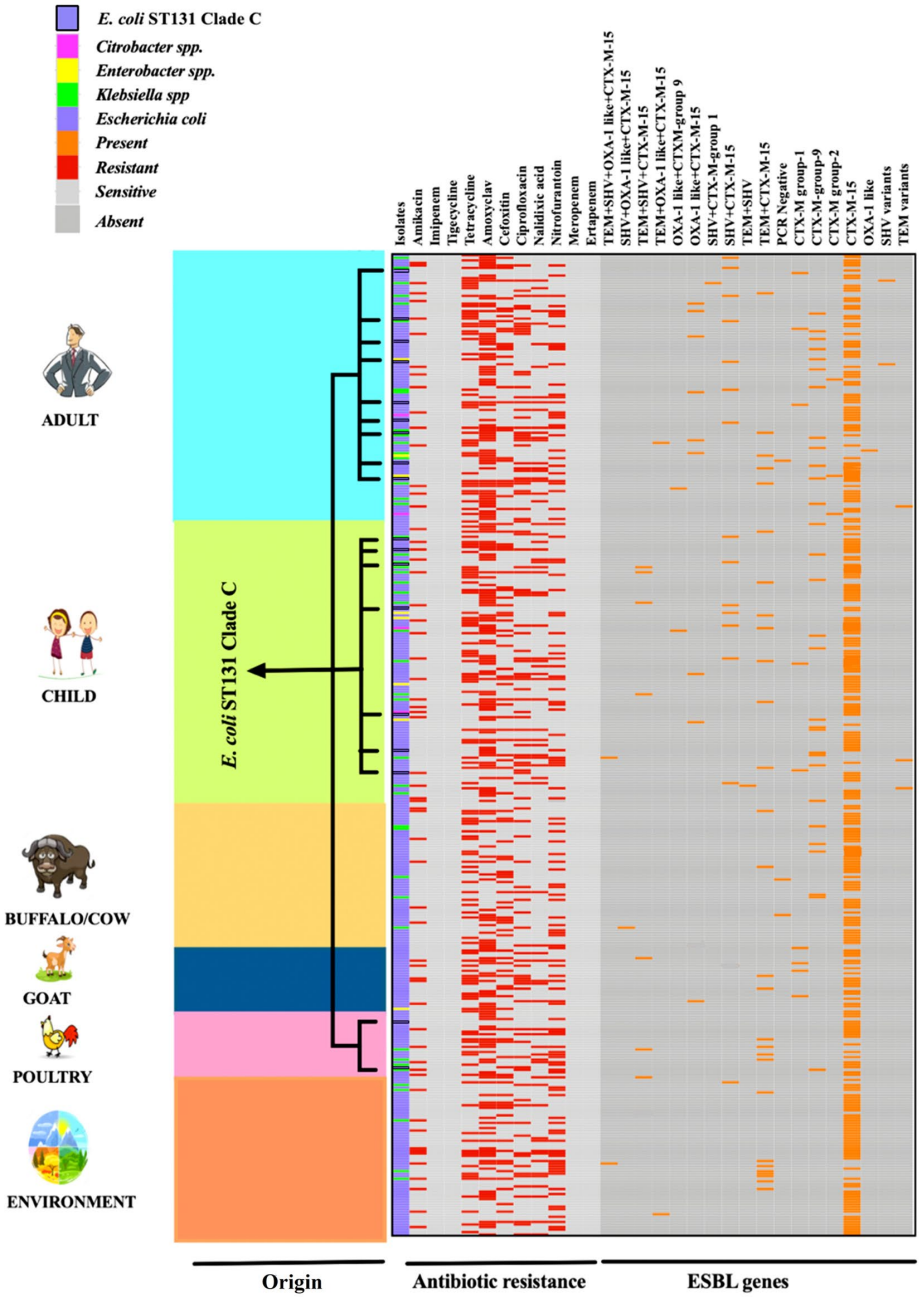
Distribution of ESBL-producing *Enterobacteriaceae* isolates, antimicrobial-resistance pattern, ESBL-associated genes, and *E. coli* ST131 high-risk clones in 128 subsistence farming families (humans, livestock, and their surrounding environment) of western Nepal. TEM: TEM variants including *bla*_TEM-1 and TEM-2_; SHV: SHV variants including *bla*_SHV-1_; *OXA-1 like:* *bla*_OXA-1,_ *bla*_OXA-4,_ and *bla*_OXA-30_; CTX-M group 1 = variants of CTX-M group 1 (includes *bla*_CTX-M-1/3,_ excluding *bla*_CTX-M-15);_ CTX-M group 2: variants of CTX-M group 2 (includes *bla*_CTX-M-2_); CTX-M group 9: variants of CTX-M group 9 (includes *bla*_CTX-M-9/14_). (Image Source: Images are downloaded from publicly available clipart: adult clipart from https://webstockreview.net/pict/getfirst; child from ; vector illustration of African buffalo cartoon by sararoom, https://depositphotos.com/29045655/stock-illustration-african-buffalo-cartoon.html; goat from https://www.freepik.com/premium-vector/cute-little-happy-goat-cartoon-vector-illustration_4852092.htm; poultry from https://www.hiclipart.com/free-transparent-background-png-clipart-plftd0; environment from https://www.123rf.com/visual/search/84518853).

Out of 174, ESBL-producing *E. coli* isolates from humans, 16 (9.2%) [adults = 9 and child = 7] belonged to ST131 and were of clade C. Out of 100 ESBL-producing *E. coli* isolates from animals, only two *E. coli* from poultry were positive for ST131 and were of clade C [Buffalo/Cow = 00, Goat = 00, Poultry = 2/35 (5.7%), Environment = 00]. Neither ST131 clade A nor clade B was detected in ESBL-producing *E. coli* strains of all groups. All the *E. coli* ST131 clade C isolated from the adult, child, and poultry carried *bla*_CTX-M-15_ (Figs. 3, 4).

## Discussion

Antibiotic resistance is recognized as a complex public health challenge because health risks arise from the resistant bacteria and their mobile genetic element transfer among humans, animals, and the environment through multiple interfaces with subsequent global dissemination^16,17^. Therefore, an international movement called One-Health is developed for an integrative approach to work in a sustainable way to inscribe the health risks at the human-animal-ecosystem interlinks^18^. Especially in LMICs, the emergence and re-emergence of disease by resistant bacteria are mainly due to poor sanitation, close interactions with livestock, climate change, easy access and irrational use of antibiotics, human behavior changes, unhygienic food preparation, and consumption practices^19^. The prevalence of ESBL-producing *Enterobacteriaceae* and CRE gut colonization in healthy humans and animals and their surrounding environment have been described previously in many regions of the world, particularly in developed countries^15^. Most of these studies were on commercial farm animals and their owners, which demonstraedt the high prevalence of AMR in conventional farming. However, such studies on organic feed livestock were sparse. This is the first one-health study investigating the occurrence of ESBL-producing *Enterobacteriaceae* and CPE in the gut of subsistence farming communities of rural areas of western Nepal.

About 85 percent of the 26 million people of Nepal live in rural areas and are engaged in farming. The majority of farming is subsistent in nature, and the crop is mostly integrated with livestock (cattle, goat, pig, and poultry). The selling of livestock products in the local market is one of the crucial income sources of farm households. The subsistence-based agricultural community often includes local breeds' of organically raised farm animals and birds. The feed used usually is of homegrown cereals and grasses. This livestock is rarely exposed to antibiotics via food additives or treatments. In this study, a high proportion (57%) of ESBL-producing *Enterobacteriaceae* was found to be colonized in the gut of subsistence farmers and their children and organically raised livestock and their surrounding environment. Furthermore, the high rate of drug resistance among organic feed livestock (43.9%) is surprising because of the limited or no use of antibiotics among subsistence farm animals, which should be considered a severe public health threat.

The exact reasons for the high ESBL-producing *Enterobacteriaceae* gut carriage rate among subsistence farming communities in this region are unknown. Still, it can be speculated that poor sanitation, low socioeconomic status, close contact with animals, and, more importantly, environmental pollution might be the reason of high carriage rate of ESBL-producing *Enterobacteriaceae* in our study groups. Besides, selective pressures of antimicrobials present in healthcare and community settings, and movement of such genes by mobile genetic elements responsible for intracellular (insertion sequence elements, transposons, and integrons) and intercellular (plasmids and integrative conjugative elements) mobility can facilitate the emergence and dissemination of AMR^20^.

Healthy subjects of the community are a vital reservoir for ESBLs, and global surveillance studies showed that the colonization rate was found to be increasing by approximately > 5% annually^7,21^. The rates are higher in the West Pacific (46%), Southeast Asia (22%), African (22%), and the eastern Mediterranean (15%)^21^. Worryingly, high rates of > 50% of ESBL-producing *E. coli* and *Klebsiella* spp. have been reported in some regions of Asia, Africa, and Latin America^22^. As stated in our previous study, the high rate of ESBL colonization (53.4%) with predominant genotype CTX-M-15 was found in healthy subjects of the rural community in Western Nepal^23^. Still, in the present study, it is striking to note that 77.6%, 43.9%, and 48% ESBL-producing *Enterobacteriaceae* with the predominant *bla*_CTX-M_ gene were observed among the gut of healthy humans, their reared animals, and the environment, respectively. Such a high percentage of ESBL-producing *Enterobacteriaceae* colonization in community settings underlines the risk to human and animal health due to disseminating resistant bacteria or their resistance genes via foodborne transmission or environmental routes, such as farm waste. These ESBL-producing *Enterobacteriaceae* isolates were susceptible to tigecycline and carbapenem only. No carbapenem-resistant or carbapenemase-producing *Enterobacteriaceae* was isolated in our study. In China, 2.4% of healthy people harbored CRE as enteric microbiota^24^. Similarly, Li et al. (2019) recently reported the presence of New Delhi Metallo-beta-lactamase type carbapenem-resistant *E. coli* (NDM-EC) among humans and backyard animals. Their study was the first to report the direct transmission of NDM-EC between humans and animals^25^.

In Nepal, the most common antibiotics used in animal sectors are tetracyclines, sulfa drugs, macrolides, polymyxins, bacitracin, nitrofurans, quinolones, and aminoglycosides, whereas chloramphenicol is the least antibiotic consumed in the veterinary sector. The rate of cotrimoxazole-resistant *E. coli* isolates from buffalo meat was reported as high as 79.6%, whereas chloramphenicol-resistant *E. coli* isolates from the milk sample were only 26%^26^. On the other hand, cotrimoxazole, amoxicillin, ciprofloxacin, chloramphenicol, nalidixic acid, gentamicin, and cephalosporins are commonly used in human health sectors^27^. In this study, higher than 50% [goat (61.5%) followed by poultry (60%), adult (56.2%), child (55%) and environmental (52.3%)] of ESBL-producing *Enterobacteriaceae* isolated from each subject showed an almost similar pattern of MDR with the highest resistance towards amoxicillin/clavulanic acid followed by tetracycline and ciprofloxacin as demonstrated in Fig. 2. The healthy children in this study had a similar fecal carriage rate of MDR isolates, as in a previous study reported from Bangladesh^28^. However, the rate of MDR in healthy adult humans was comparatively higher than that of studies previously conducted in Nepal^29^. The MDR bacterial colonization in animal and environmental isolates are comparatively lower in this study than that of a survey carried out in Nigeria^30^ and India^31^. A single study determining the rate of MDR in all domains of one-health like environment, healthy humans and animals is limited. In this study, the ESBL-producing *Enterobacteriaceae* isolates of humans showed the highest (63.1%) resistance towards amoxicillin/clavulanic acid, and goat isolates showed 47% resistance towards tetracycline. The environmental strains showed 40.5% resistance towards nitrofurantoin. The highest resistance rate towards ciprofloxacin was observed in the ESBL-producing *Enterobacteriaceae* of goat isolates (34.5%). The rest of the antibiotic resistance rates by all three one-health domains showed less than 30%. The occurrence of combined resistance towards ampicillin, tetracyclines, and cotrimoxazole has become common in ESBL isolates as the genes encoding resistance to these antibiotics are located on the same plasmid^32^.

Farmers in rural areas of Nepal are still following traditional farming practices and are less likely to implement proper management and hygiene practices^26^. The irrational use of antibiotics to reduce mortality due to infection in cattle, pigs, and poultry without prior consultation of a veterinarian^26^ is customary and widespread among conventional farming in Nepal. Appropriate and safe use of antibiotics in livestock is rarely practiced^25^. Subsistent farmers commonly use untreated manure as fertilizer or for bedding and irrigation of agricultural lands, thus discharging antimicrobial residues, resistant bacteria, and genes into the environment. Besides, frequent contact with a wide variety of animals without standard disinfection and hygiene procedures may lead to MDR-strains transmission to humans^33^.

The main drivers of AMR development in humans are self-medication, over-prescription, under-prescription, syndromic management approaches, and irrational prescription of powerful antibiotics for a speedy cure, lack of well-equipped hospitals and clinics^1,23,26^. In our study, rectal samples of healthy subjects and environmental samples were collected after the disastrous earthquake struck in Nepal in 2015. The alarming rate of ESBL-producing *Enterobacteriaceae* (57%) in this study might be influenced by post-earthquake scenario. There was a dramatic increase in water-borne diseases and the destruction of irrigation and drainage canals, releasing more pathogenic strains into the environment. Despite considering the factors influenced by a natural disaster, the percentage of healthy subjects carrying ESBL-producing *Enterobacteriaceae* strains in our community was comparable to that of our neighboring countries; 68% in India^34^, up to 52% in Pakistan^35^, and 50% in China^25^.

The global picture of variant CTX-M is complex, but it is evident that *bla*_CTX-M-15_ has increased over the years and is dominant in most regions. The fecal carriage rate of CTX-M-15 is prevailing in Asia, whereas group 9 variants, especially CTX-M-14 in China, South East Asia, South Korea, Japan, and Spain, and CTX-M-2 in South America, remain significant^36^. CTX-M-15 type ESBL-producing *Enterobacteriaceae* reservoirs of environment and livestock regularly exchange clones, and mobile genetic elements with the human reservoir by transposition or transduction, leading to clonal and epidemic plasmid spread^9,36^. In this study, CTX-M-15 is found to be the dominant gene in the environment (100%), cattle (100%), poultry (100%), goat (84.6%), human adult (95.2%), and child (97.9%). In fact, the river, soil, and nearby drinking water sources of Nepal are contaminated with high levels of fluoroquinolones through wastewater effluents of pharmaceutical industries^26^. The pollution of the environment from human and animal waste, low sanitation standards, and contaminated drinking water increases the cycling of CTX-M possessing ESBL-producing *Enterobacteriaceae* between humans and the environment^36^. It can be hypothesized that reduced access to the lavatory in addition to high population density, high migration rate (remittance serves 25% of GDP, highest among South Asian countries; https://nra.org.np/nra_news/remittance-keeping-economy-afloat/) can make Nepal one of the epicenters of dissemination of ESBL-producing *Enterobacteriaceae* carrying *bla*_CTX-M-15_. The situation was similar in India, where ESBL-producing *Enterobacteriaceae* carriage rates (> 68%) were the highest in the world. Such a tremendous rate warrants the dissemination of gut microbes carrying genes such as *mcr-1* and *bla*_NDM-1_^34,36^.

The persistence of resistance in commensal *E. coli* is a significant marker for the selective pressure enforced by antibiotic use and subsequent resistance predicted in pathogens^37^. In this study, *E. coli* (75.7%) isolates harbored the highest single CTX-M-15 gene, followed by *Citrobacter* spp. (50%), *Enterobacter* spp. (44.4%) and *Klebsiella* spp. (34%). On the contrary, *Klebsiella* spp. (48.8%) possessed the highest multiple genes containing CTX-M-15, followed by *Citrobacter* spp. (25%), *Enterobacter* spp. (22.2%) and *E. coli* (11.4%). One of the prominent human AMR high-risk clones includes *E. coli* ST131 with *bla*_CTX-M_ types^20^. High-risk clone of ESBL-producing *E. coli* ST131 is dominating globally. Clade C is the most common global clade among clinical ST131 and is associated with fluoroquinolone resistance^9^. Sherchan et al.^38^ revealed that > 90% of clinical ESBL-producing *E. coli* isolates in Nepal were CTX-M-15 positive, and more than half possessed ST131. ESBL-producing *E. coli* carrying ST131 isolates of dogs and cats were first reported from a Portuguese study^39^. The *E. coli * ST131 is seldom responsible for nosocomial outbreaks^9^. The transmission mode of *E. coli* ST131 in the community setting is currently unknown^9^. Therefore, investigations regarding the roles of environmental reservoirs, companion animals, and direct or indirect person-to-person transmission are epidemiologically pertinent in the community transmission of ST131^9^. We found that 9.2% of humans (adults 9, children 7) and 5.7% of poultry ESBL-producing *E. coli* isolates carried ST131 clade C with *bla*_CTX-M-15_. The low prevalence of ST131 in non-human isolates suggests that ST131 might be originated from human sources and transmitted directly or indirectly among humans^9,40^. The study conducted by Ewers et al.^41^ found that many human and companion animals' clinical ST 131 *E. coli* shared similar virulence, resistance, plasmid content, and Pulsed-field Gel Electrophoresis (PFGE) profile. The emergence of *E. coli* ST131 is due to the widespread use of fluoroquinolones and oxyimino-cephalosporins. Compared to other Extra-intestinal Pathogenic *E. coli* (ExPEC) clones or different ST131 clades, *E. coli* ST 131 clade C is found to be inherently more adaptable in the environment, even without antimicrobial selection pressure. Moreover, a single high-risk clone of *E. coli* ST131 clade C plays a significant role in the worldwide distribution of ESBL-producing bacteria^9,20,37^.

There are rules and regulations regarding the judicious use of antibiotics and the infection control system in Nepal. However, the problem lies in implementation^42^. Such non-compliance activities and guidelines have expedited the further emergence and spread of resistant microbes^43^. To restrict AMR risk and mitigate its effect on human and animal health, a multidisciplinary approach involving public–private collaborators and government agencies is required. It is crucial to enforce initiatives such as immunization, public literacy, better hygiene standards, antibiotic stewardship, genomic surveillance program, decreased use of antibiotics in agriculture and livestock, and good husbandry practices. Moreover, proper waste segregation, disposal system, handling, transport, and medical waste treatment should be practised to mitigate the direct effects on human health and the environment. Thus, public health and environmental professionals, health care practitioners, and veterinarians should regard the 'one-health approach' as a professional imperative for the shared interests of health promotion and global tackling of antimicrobial resistance.

## Limitations

Although the study is foremost in Nepal, there are some limitations. The study lacks an extensive investigation of the different sequence types and the clonal diversity of ESBL-producing *Enterobacteriaceae* isolates. Similarly, carbapenemase genes were not amplified by genotype based methods. Moreover, all PCR products were not subjected to sequencing to affirm the ESBL variants. Therefore, intensive one-health surveillance, molecular sorting, and genomics-based research will help in understanding the significance of MDR gut flora in the dissemination of AMR and its interdependence in human and animal infections.

## Conclusion

This is the first one-health study in western Nepal, determining the high rate of CTX-M-15 type ESBL-producing *Enterobacteriaceae* among gut flora of subsistence-based farming communities. Gut colonization by *E. coli* ST131 clade C among healthy farmers and poultry birds is a significant public health problem. The wide dissemination of ESBL-producing *Enterobacteriaceae* among organically raised livestock and owners, including their children, may contribute to the shortfalls in infection control practices and public health management in western Nepal. Further implementation of AMR mitigation strategies (the framework to identify, prioritize and implement) across the one-health spectrum is essential to combat and unravel the complexities associated with the emergence, evolution, and dissemination of antimicrobial resistance in subsistence-based farm settings.

## Materials and methods

### Study design, site, and enrollment of participants

**Figure 5:**
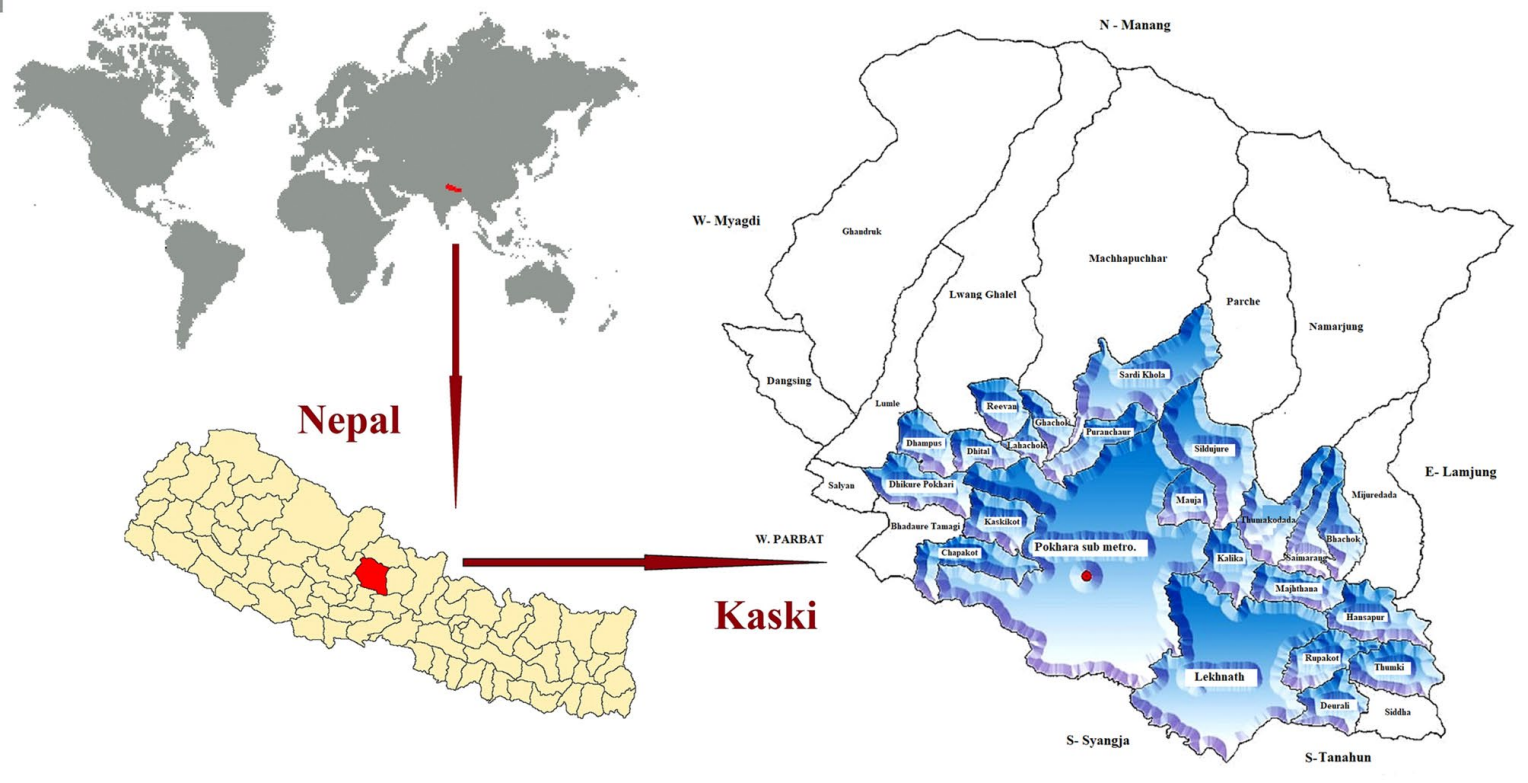
Kaski district of western Nepal, where the specimen was collected from subsistence farming communities (light blue area). Three maps are recolored versions of publicly available maps. Source: Blank world map by E Pluribus Anthony under Public Domain license, https://creativecommons.org/publicdomain/zero/1.0/ (https://commons.wikimedia.org/wiki/Maps_of_the_world#/media/File:BlankMap-World-noborders.png), Nepal is highlighted with red color. Location map of Nepal by Biplab Anand licensed under the Creative Commons Attribution-Share Alike 4.0 International https://creativecommons.org/licenses/by-sa/4.0/deed.en. (https://commons.wikimedia.org/w/index.php?title=Special:Search&limit=20&offset=20&ns0=1&ns6=1&ns12=1&ns14=1&ns100=1&ns106=1&search=nepal+map&advancedSearch-current=%7B%7D#/media/File:Nepal_districts.png), the Kaski district is highlighted with red color. Political map of Kaski District by LGCDP/MoFALD licensed under the Creative Commons Attribution-Share Alike 4.0 International https://creativecommons.org/licenses/by-sa/4.0/deed.en (https://commons.wikimedia.org/wiki/File:Political_Map_of_Kaski_District.jpg), the sites of sample collected from subsistence farming communities are highlighted with light blue color.

During the study period, rural areas of districts in Nepal were subdivided into federal entities called Village Development Committees (VDCs), and the urban or metropolitan regions were divided into wards. Nepal was divided into 14 zones, and the study area Kaski district was under the Gandaki zone (Fig. 5). The Kaski district was divided into 47 VDCs and two metropolitan areas (Pokhara and Lekhnath), with a total population of 492,098 (2011 census). The population in each VDC was around 3,000–12,000. Based on statistical power calculations and the expected farming communities in the Kaski district, our goal was to obtain 125 subsistence farming families. All 47 VDCs were numbered according to alphabetic order; 23 (49%) VDCs were randomly selected applying the formula = RANDBETWEEN (1, 47). The random numbers procreated were 1, 2, 4, 15, 40, 27, 16, 33, 31, 5, 22, 17, 37, 26, 45, 34, 23, 9, 41, 39, 8, 13, and 28. The study area of the Kaski district is demonstrated in Fig. 5. Houses were randomly selected from the 2008 voter registration list provided by the Nepal Government Electoral Commission. The recruitment of subsistence farmers' houses was made by visiting the residences in the selected VDCs who fulfilled the inclusion criteria. During the study period (May 2016 to December 2018), a rectal swab specimen was collected from healthy subjects of 128 houses involved in subsistence farming who volunteered to participate in the study. On average, five houses were selected per VDC. The subsistence farming communities having one adult and one child and a minimum of one livestock were involved in specimen collection. Organic fed pasture-raised animals and their owners and children without a history of antibiotic consumption or hospitalization during the last three months at the time of sample collection were included in the study. The exclusion criteria included; participants who refused to provide samples and consent, a history of hospitalization (< 3 months), antibiotic consumption (< 3 months), family members working in hospitals, and the presence of unhealthy animals. The selected residences, where either owner or livestock was not available during the visit for sample collection, were also excluded.

### Specimen collection and questionnaire

#### The rectal swab from human, livestock and environmental specimens

Sterile cotton swab (HiMedia Laboratories, India) pre-moistened with sterile normal saline was inserted into 1–1.5 inches deep into the rectum and gently rotated for a few seconds. After specimen collection, the swabs were inoculated in a tube containing 0.1% peptone water (HiCulture Transport Swab, HiMedia, India). A cloacal swab from chickens was taken by inserting a swab into the vent and by gently swabbing the mucosal wall till the swab was stained with fecal material. For environmental specimens, the swab specimens were collected from the drainage or sewage area near each selected house. All samples were transported to the Microbiology Laboratory of Manipal Teaching Hospital in specimen transport containers with ice packs. Based on the distance between the laboratory and the place of a visit, the samples were processed within a maximum of 8 h of collection. Detailed questionnaires on demographic and husbandry practices were included.

### Detection of ESBL-producing *Enterobacteriaceae* and CRE

#### Screening for ESBL-producing *Enterobacteriaceae* from rectal swabs

Each swab was placed in a tube containing 1 ml of sterile 0.9% saline and vortexed for 30 s. For ESBL-producing *Enterobacteriaceae* screening, 100 µl of the suspension was inoculated on commercial ESBL-selective chromogenic medium (HiMedia, India) and that for carbapenem-resistant *Enterobacteriaceae* on MacConkey agar with 1 μg/ml imipenem (HiMedia, India). The plates were incubated at 37 °C for 24 h under aerobic conditions. The color of the colonies was noted according to the manufacturer's color chart (Pink = *E. coli*, Blue = *Klebsiella* spp., Colourless = other Gram-Negative Bacilli) for presumptive identification of ESBL-producing *Enterobacteriaceae* isolates (supplementary file 3: Fig. S2). A single isolated colony of each color was sub-cultured on Nutrient Agar (HiMedia, India) for phenotypic identification and preservation^44^. All presumptive ESBL-positive isolates were preserved in Brain Heart Infusion (HiMedia, India) glycerol broth in stock vials at -20^0^C for further study.

### Phenotypic confirmations of ESBL and CRE screen positive isolates

#### ESBL detection by double-disc synergy test

The presumptive ESBL-positive isolates were retested for ESBL production by the Double Disc Synergy Test (DDST)^45^. Set of two discs containing extended-spectrum cephalosporin [cefotaxime (30 μg) or ceftazidime (30 μg) alone and with a clavulanic acid combination (10 μg) were placed on-center spacing 25 mm apart on a Mueller Hinton Agar (HiMedia, India) plates inoculated with a bacterial suspension compared with 0.5 McFarland turbidity standard. Zone diameters were measured after overnight incubation at 37 °C. Strains resistant to cefotaxime (zone diameter ≤ 27 mm) or ceftazidime (zone diameter ≤ 22 mm) and an increase in zone diameter ≥ 5 mm with the discs containing clavulanic acid was defined as ESBL-producing isolates (Supplementary file 4: Fig. S3).

#### Carbapenemases detection by phenotypic methods

For carbapenemase detection, all ESBL isolates were screened phenotypically by Modified Hodge Test (MHT)^46^ and modified Carbapenem Inactivation Method (mCIM)^47^ as described elsewhere. For the modified Hodge test, a 0.5 McFarland dilution of the *Escherichia coli* ATCC 25922 in 5 ml of normal saline was prepared. A 1:10 dilution was streaked as a lawn on MHA. A 10 µg meropenem, imipenem, or ertapenem disc (MASTDISCS, U.K) was placed in the center of the test area. From the edge of the disc to the edge of the plate, the test organism was streaked in a straight line. The plate was incubated overnight at 37 °C. MHT Positive *Klebsiella pneumoniae* ATCC1705 and MHT Negative *Klebsiella pneumoniae* ATCC1706 were used with each batch of the test. A clover leaf-like indentation at the intersection of the *E. coli* 25922 and the test organism within the inhibition zone of the carbapenem disc interpreted as positive MHT while no growth of the *E. coli* 25922 along with the growth streak of test organism within the disc as negative MHT.

For mCIM method, one loop of a fresh colony of the strain was inoculated in the tube containing 2 ml of Trypticase Soy Broth (TSB) (HiMedia, India) using 1 μL calibrated inoculating loop. A 10 μg meropenem disc was incubated in a suspension of tested strain for 4 h at 35 °C in ambient air. Then, the meropenem disc was drained from TSB suspension using a 10 μL inoculating loop and placed on the Mueller Hinton Agar plates inoculated with 0.5 McFarland suspension of *E. coli* ATCC 25922 following the routine disk diffusion procedure. After overnight incubation, a zone diameter of 6–15 mm, or 16–18 mm with small colonies in the inhibitory zone of meropenem was considered positive. A zone size of meropenem ≥ 19 mm was considered carbapenemase negative. A zone diameter of 16-18 mm was considered as intermediate result (required a further test to confirm the presence or absence of carbapenemase production).

### Antimicrobial susceptibility testing

The antibiotic susceptibility profiles of the ESBL-positive isolates were determined using a panel of antibiotics of human and veterinary clinical pertinence by Kirby-Bauer disk diffusion method as per CLSI guidelines^48^ and interpreted based on CLSI 2016 and 2017 breakpoints^49^. FDA breakpoint (http://www.accessdata.fda.gov/drugsatfda_docs/label/2009/021821s016lbl.pdf) was used for the interpretation of tigecycline. One or two representatives from various classes of antibiotics (MASTDISCS, U.K) were included: co-amoxiclav (beta-lactam combination agents), cefotaxime and ceftazidime (3rd generation cephalosporins), cefoxitin (2nd generation cephalosporins), tetracycline (tetracyclines), amikacin (aminoglycosides), nalidixic acid, ciprofloxacin (fluoroquinolones), nitrofurantoin (nitrofurans), tigecycline (glycylcycline) and imipenem, meropenem and ertapenem (carbapenems). *E. coli* ATCC 25922 was used as quality control strain.

### Genotypic screening of ESBL phenotypes

Phenotypically ESBL-positive isolates were screened for ESBL encoding genes by multiplex PCR. As per Dallenne C et al.^50^, two multiplex PCR were assayed to detect the presence of β-lactamase genes *bla*_TEM_/*bla*_SHV_/*bla*_OXA-1_ and *bla*_CTX-M,_ including phylogenetic groups 1, 2, and 9. The primers (Sigma-Aldrich) used, and the size of the expected DNA products for each enzyme group are shown in supplementary file 5: Table S3. The commercially available DNA purification kit (HipurA bacterial genomic DNA purification Kit, HiMedia, India) was used for DNA extraction. 25 μL of PCR reactions (2X Amplicon Red Taq master mixes consisting of Tris HCl P^H^ 8.5, (NH_4_)_2_ SO_4_, 4 mM MgCl_2_, 0.2% Tween 20, 0.4 mM dNTPs, and 0.2 units/μL amplicon Taq DNA polymerase) was carried out. 0.2 μM to 0.4 μM primer and 1 μL template DNA were used (supplementary file 5: Table S3). Cycling protocol was as follows: initial denaturation at 94 °C for 10 min; 30 cycles of 94 °C for 40 s, 60 °C for 40 s and 72 °C for 1 min; and a final elongation step at 72 °C for 7 min. Phylogroup-1-positive isolates were further characterized for *bla*_CTX-M-15_ gene by uniplex PCR assay^51^. Amplification was performed with 10 μL 2X Amplicon Red Taq master mixes; 1 μl forward and reverse primer and 1 μL purified DNA in a total volume of 25 μL. The CTX-M-15 positive isolates were confirmed bidirectionally by sequencing. Bioedit V.7.2.1 software was used for aligning obtained sequences, and consensus sequences were compared with CTX-M-15 gene sequences in the Genbank database through the Basic Local Alignment Search Tool (BLAST) program (http://www.ncbi.nlm.nih.gov/BLAST).

### Screening for *E. coli* ST131 clades

Sequence type-131 clonal lineage and ST131 clades (A, B, and C) of all ESBL-positive *E. coli* isolates were screened by multiplex PCR as described by Matsumura et al.^52^. After running at 100 V for one hour on 2% agarose gel containing ethidium bromide, amplicons were visualized. A size marker of 100 bp DNA ladder (Eurofins Scientific, India) was used. For positive and negative controls, known *E. coli* ST131 and non-ST131 *E. coli* were used, respectively. The size of the expected DNA products and the primers used in this study for each enzyme group are listed in supplementary file 5: Table S3.

### Ethics approval and consent to participate

The research proposal was approved by the Institutional Ethics Committee, Manipal Teaching Hospital, Pokhara, Nepal. Reference number: MEMG/IRC/GA/1269/2015. The study was conducted in compliance with the latest version of the Declaration of Helsinki. All methods were carried out in accordance with relevant guidelines and regulations. This study was carried out in compliance with the ARRIVE guidelines approved by MCOMS, Nepal and MAHE, India.

### Statistical analysis

Significant differences in the prevalence and high degree of antibiotic resistance between different populations were assessed by the Epi-info and SPSS software. GraphPad Prism Version 8.1.2 (227) was used to develop a heat map showing an antibiotic resistance profile and ESBL genes.

### Consent for publication

Written informed consent was obtained from each individual participating voluntarily in the study.

## Supplementary Information

Supplementary Information

## Abbreviations

MDR: Multi-drug resistant/resistance
ESBL: Extended-spectrum β-lactamase
CPE: Carbapenemase-producing *Enterobacteriaceae*
PCR: Polymerase chain reaction
MLST: Multilocus sequence typing
VDCs: Village development committees
AmpC: Ambler class C
IQR: Interquartile range
DDST: Double disc synergy test
CLSI: Clinical & Laboratory Standards Institute
ATCC: American type culture collection
