# Supplementary material for: Detection and characterization of ESBL-producing Enterobacteriaceae from the gut of subsistence farmers, their livestock, and the surrounding environment in rural Nepal
Source: Sci Rep. 2021 Jan 22;11:2091. doi: 10.1038/s41598-021-81315-3 (PMC7822894; doi:10.1038/s41598-021-81315-3)
Supplement: Supplementary file 1 — Supplementary Information [file 41598_2021_81315_MOESM1_ESM.pdf]

Supplementary files

Detection and Characterization of ESBL-producing *Enterobacteriaceae* from the Gut of Subsistence Farmers, their Livestock and Surrounding Environment in Rural Nepal

Supram Hosuru Subramanya (supram.gowda@gmail.com)[1]*, Indira Bairy[2] (bairyindira@gmail.com), Yang Metok[1] (yangmetok@gmail.com), Bharat Prasad Baral[1] (bharatprasad999baral@gmail.com), Dipendra Gautam[1], (dgtm420@gmail.com), Niranjan Nayak[1] (niruni2000@yahoo.com).

[1]Manipal College of Medical Sciences, Pokhara, Nepal, [2]Melaka Manipal Medical College, Manipal Academy of Higher Education, Manipal, India.

Supplementary files:

1. Supplementary file 1: Fig. S1: Stacked bar graph showing multidrug resistance pattern of the ESBL-producing *Enterobacteriaceae* isolates of different study groups.

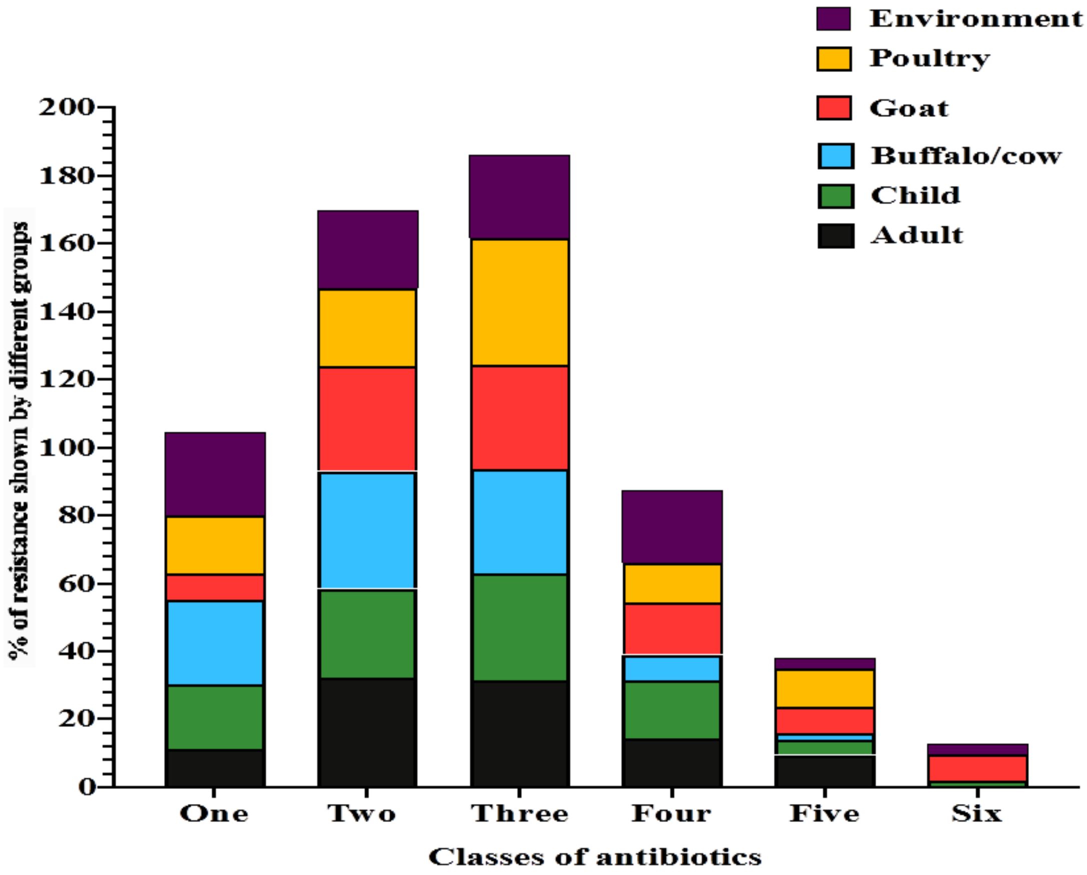

2. Supplementary file 2:Table S1: Summary of ESBL genes identified in six groups of subsistence farming communities

ESBL genes	Adult	Child	Buffalo/ Cow	Goat	Poultry	Environment
	N=105 (%)	N=111 (%)	N=52 (%)	N=26 (%)	N=35 (%)	N=65 (%)
**Single Gene**	83(79)	88(79.3)	47(90.4)	23(88.4)	29(82.9)	53(81.5)
bla$_{\mathbf{TEM}}$	01(0.95)	02(1.8)	00	00	00	00
bla$_{\mathbf{SHV}}$	02(1.9)	00	00	00	00	00
bla$_{\mathbf{OXA\text{-}1\ like}}$	01(0.95)	00	00	00	00	00
**CTX-M group 1 (except *bla*$_{\mathbf{CTX\text{-}M\text{-}15}}$)**	03 (2.8)	02(1.8)	00	04(15.4)	00	00
**CTX-M group 2**	03(2.8)	00	00	00	00	00
**CTX-M group 9**	12(11.4)	11(9.9)	06(11.5)	00	01(2.9)	00
bla$_{\mathbf{CTX\text{-}M\text{-}15}}$	61(58.1)	73(65.8)	41 (78.8)	19(73.1)	28(80)	53(81.5)
**Multiple genes**	21(20)	23(20.7)	03(5.8)	03(11.5)	06(17.1)	12(18.5)
bla$_{\mathbf{TEM}}$**+ *bla***$_{\mathbf{SHV}}$**+*bla***$_{\mathbf{CTX\text{-}M\text{-}15}}$	00(00)	04(3.6)	00(00)	00(00)	01(2.9)	02(3.1)
bla$_{\mathbf{TEM}}$ **+ *bla***$_{\mathbf{CTX\text{-}M\text{-}15}}$	05(4.7)	07(6.3)	02(3.8)	02(7.7)	04(11.4)	08(12.3)
bla$_{\mathbf{TEM}}$ **+ *bla***$_{\mathbf{SHV}}$ **+ *bla***$_{\mathbf{OXA\text{-}1\ like}}$ **+ *bla***$_{\mathbf{CTX\text{-}M\text{-}15}}$	00(00)	01(0.9)	00(00)	00(00)	00(00)	01(1.5)
bla$_{\mathbf{OXA\text{-}1\ like}}$ **+ *bla***$_{\mathbf{CTX\text{-}M\text{-}15}}$	06 (5.7)	02(1.8)	00(00)	01(3.8)	00(00)	00(00)
bla$_{\mathbf{TEM}}$**+ *bla***$_{\mathbf{OXA\text{-}1\ like}}$ **+ *bla***$_{\mathbf{CTX\text{-}M\text{-}15}}$	01(0.95)	00 (00)	00(00)	00(00)	01(2.9)	00(00)
bla$_{\mathbf{SHV}}$**+ *bla***$_{\mathbf{OXA\ like}}$ **+ *bla***$_{\mathbf{CTX\text{-}M\text{-}15}}$	00 (00)	00(00)	01(1.9)	00(00)	00(00)	00(00)
bla$_{\mathbf{OXA\text{-}1\ like}}$**+ CTX-M group 9**	01(0.9)	01(0.9)	00(00)	00(00)	00(00)	00(00)
bla$_{\mathbf{SHV}}$**+ *bla***$_{\mathbf{CTX\text{-}M\text{-}15}}$	07(6.6)	07(6.3)	00(00)	00(00)	00(00)	01(1.5)

***bla*$_{SHV}$+ CTX-M group 1**	01(0.9)	00(00)	00(00)	00(00)	00(00)	00(00)
***bla*$_{TEM}$ + *bla*$_{SHV}$**	00(00)	01(0.9)	00(00)	00(00)	00(00)	00(00)
PCR negative	01(0.9)	00(00)	2(3.8)	00(00)	00(00)	00(00)

Table S2: Summary of ESBL genes identified in 394 ESBL producing isolates

**ESBL genes**	**Total N (%)**	***E. coli* N (%)**	***Klebsiella* spp. N (%)**	***Enterobacter* spp. N (%)**	***Citrobacter* spp. N (%)**
TEM+SHV+OXA-1 like+CTX-M-15	2 (0.5)	1 (0.3)	1 (2.1)	0 (0)	0 (0)
SHV+OXA-1 like+CTX-M-15	1 (0.3)	0 (0)	1 (2.1)	0 (0)	0 (0)
TEM+SHV+CTX-M-15	7 (1.8)	2 (0.6)	5 (10.6)	0 (0)	0 (0)
TEM+OXA-1 like+CTX-M-15	2 (0.5)	1 (0.3)	1 (2.1)	0 (0)	0 (0)
OXA-1 like + CTX-M-group 9	2 (0.5)	1 (0.3)	1 (2.1)	0 (0)	0 (0)
OXA-1 like + CTX-M-15	9 (2.3)	6 (1.8)	3 (6.4)	0 (0)	0 (0)
SHV + CTX-M group 1	1 (0.3)	0 (0)	1 (2.1)	0 (0)	0 (0)
SHV + CTX-M-15	15 (3.8)	7 (2.1)	7 (14.9)	1 (11.1)	0 (0)
TEM + SHV	1 (0.3)	0 (0)	1 (2.1)	0 (0)	0 (0)
TEM + CTX-M-15	28 (7.1)	21 (6.3)	5 (10.6)	1 (11.1)	1 (25)
CTX-M group 1	9 (2.3)	9 (2.7)	0 (0)	0 (0)	0 (0)
CTX-M group 9	30 (7.6)	23 (6.9)	5 (10.6)	2 (22.2)	0 (0)
CTX-M group 2	3 (0.8)	1 (0.3)	0 (0)	1 (11.1)	1 (25)
CTX-M-15	275 (69.8)	253 (75.7)	16 (34)	4 (44.4)	2 (50)
OXA-1 like	1 (0.3)	1 (0.3)	0 (0)	0 (0)	0 (0)
SHV-variants	2 (0.5)	2 (0.6)	0 (0)	0 (0)	0 (0)
TEM-variants	3 (0.8)	3 (0.9)	0 (0)	0 (0)	0 (0)
PCR Negative	3 (0.8)	3 (0.9)	0 (0)	0 (0)	0 (0)
**Total**	**394**	**334**	**47**	**9**	**4**

Legend: TEM: TEM variants including *bla*$_{TEM-1 \text{ and } TEM-2}$**; SHV:** SHV variants including *bla*$_{SHV-1}$**; OXA-1 like**: *bla*$_{OXA-1}$, *bla*$_{OXA-4}$, and *bla*$_{OXA-30}$; **CTX-M group 1**= variants of CTX-M group 1 (includes *bla*$_{CTX-M-1}$ and *bla*$_{CTX-M-3}$, except *bla*$_{CTX-M-15}$); **CTX-M group 2**: variants of CTX-M group 2 (includes *bla*$_{CTX-M-2}$); **CTX-M group 9**: variants of CTX-M group 9 (includes *bla*$_{CTX-M-9/14}$).

3. Supplementary file 3: Fig. S2: Rectal swab cultured on ESBL HiChrom agar: (A) Pink colonies of *E. coli*. (B) Blue colonies of *Klebsiella* spp. (C) The mixture of *E. coli* (pink) and *Klebsiella* spp. (blue).

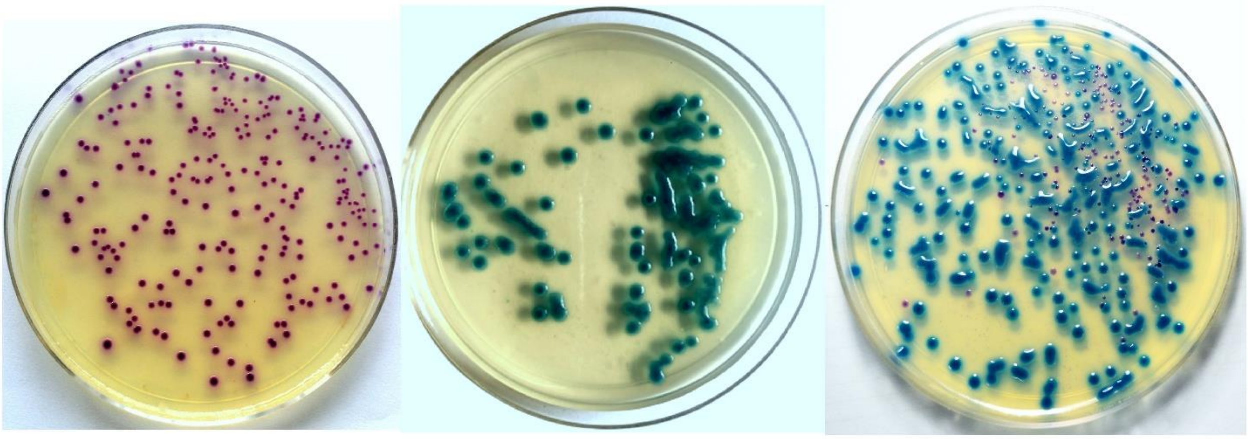

4. Supplementary file 4: Fig. S3:A-The double-disc synergy test: A-An increase in the inhibition zone diameter of >5 mm for ceftazidime + clavulanic acid (30 µg/10 µg) versus ceftazidime disc (30 µg) alone confirmed ESBL production. **B-** Antibiotic susceptibility by Kirby-Bauer disk diffusion method.

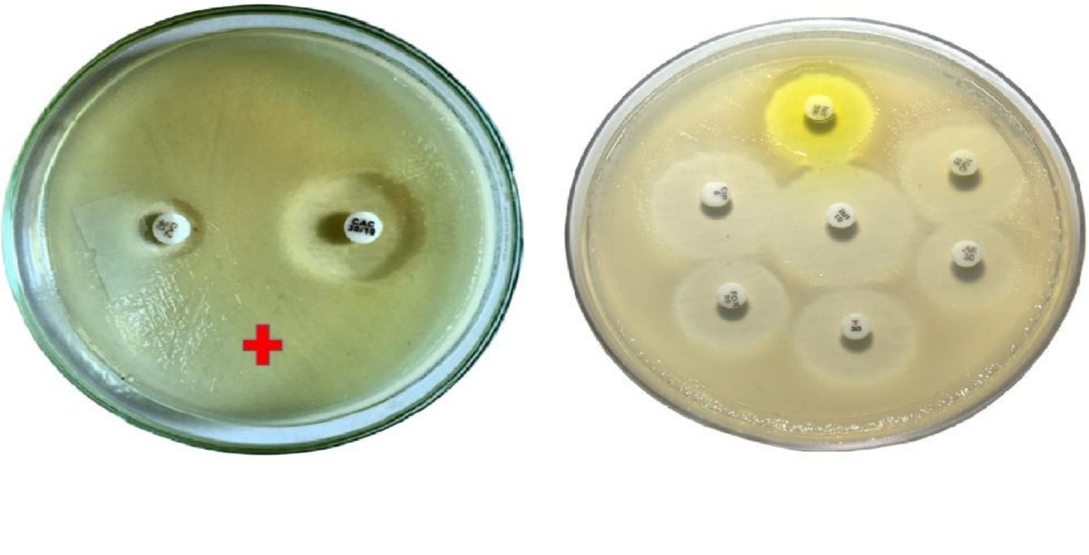

5. Supplementary file 5: Table S3: List of primers used for gene amplification.

Sl. no	PCR name	β-lactamase(s) targeted	Sequence (50 –30)	Length (bases)	Amplicon size (bp)	Primer concentration (pmol/mL)	Reference
1	**Multiplex I** TEM, SHV and OXA-1-like	**TEM variants** including TEM-1 and TEM-2	F-CATTTCCGTGTCGCCCTTATTC<br>R-CGTTCATCCATAGTTGCCTGAC	22<br>22	800	0.4<br>0.4	51
		**SHV variants** including SHV-1	F-AGCCGCTTGAGCAAATTAAAC<br>R-ATCCCGCAGATAAATCACCAC	21<br>21	713	0.4<br>0.4	
		**OXA-1, OXA-4 and OXA-30**	F-GGCACCAGATTCAACTTTCAAG<br>R-GACCCCAAGTTTCCTGTAAGTG	22<br>22	564	0.4<br>0.4	
2	**Multiplex II** CTX-M group 1, group 2 and group 9	**Variants of CTX-M group 1** including CTX-M-1, CTX-M-3 and CTX-M-15	F-TTAGGAARTGTGCCGCTGYAb<br>R-CGATATCGTTGGTGGTRCCAT[b]	20<br>21	688	0.4<br>0.2	51
		**Variants of CTX-M group 2** including CTX-M-2	F-CGTTAACGGCACGATGAC<br>R-CGATATCGTTGGTGGTRCCAT[b]	18<br>21	404	0.2<br>0.2	
		**Variants of CTX-M group 9 including CTX-M-9 and CTX-M-14**	F-TCAAGCCTGCCGATCTGGT<br>R-TGATTCTCGCCGCTGAAG	19<br>18	561	0.4<br>0.4	
3	**Singleplex**	**CTX-M-15**	F-CACACGTGGAATTTAGGGACT<br>R-GCCGTCTAAGGCGATAAACA	21<br>20	996	0.1<br>0.1	52
5	**Multiplex IV**<br>**ST131 clade**	**ST131**	F-AGCAACGATATTTGCCCATT<br>R-GGCGATAACAGTACGCCATT	20<br>20	580	0.15<br>0.15	53
		**Clade A**	F-TGACGGGACGTGAGCAAATTA<br>R-AGTCAGACCTAGCCACCCTT	21<br>20	707	0.15<br>0.15	
		**Clade B**	F-CAACGTTGAAGCAGTGTATGAG<br>R-TGACAATCGACGGCTTTAGA	22<br>20	442	0.08<br>0.08	
		**Clade C**	F-CGCTGGCCAGTTATCTGAAAT<br>R- CCTTTCACCAACTGGGTTACT	21<br>21	103	0.2<br>0.2	

Legend: [b]**Y**=T or C; **R**=A or G.

6. Supplementary file 6: Fig. S4: Agarose gel electrophoresis of CTX-M-15 PCR Lane M, 100-bp DNA ladder; lane 1- negative control; lane 2- positive control; lane 3 to19- test strain positive for CTX-M-15 (996 bp).

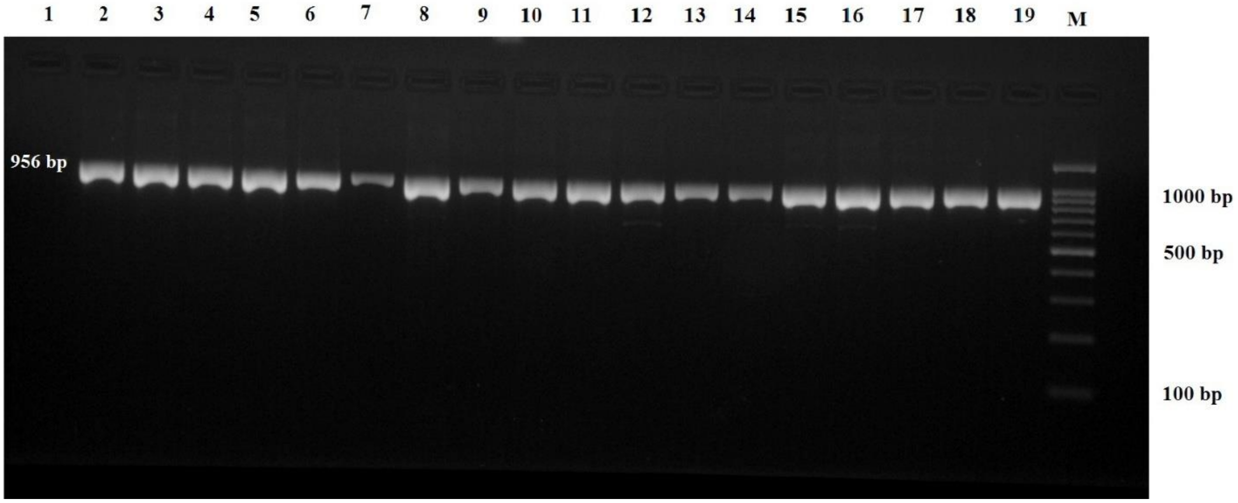

7. Supplementary file 7: Table S4: Antibiotic resistance pattern of ESBL-producing *Enterobacteriaceae* strains isolated from different groups of the community

	Amikacin (%)	Imipenem (%)	Tigecycline (%)	Tetracycline (%)	Amoxyclav (%)	Cefoxitin (%)	Ciprofloxacin (%)	Nalidixic acid (%)	Meropenem (%)	Ertapenem (%)	Nitrofurantoin (%)
**Adult (105)**	15.5	0	0	41.8	63.1	26.3	33	20.4	0	0	29.2
**Child (111)**	15.3	0	0	36.9	56.8	27	29.7	22.5	0	0	25.8
**Buffalo/ Cow (52)**	11.6	0	0	33.2	28.2	29.8	14.9	1.7	0	0	26.6
**Goat (26)**	17.2	0	0	47	55.7	28.8	34.5	22.1	0	0	31.6
**Poultry (35)**	14.7	0	0	36.8	55.3	25.8	22.1	22.1	0	0	40.5
**Environment (65)**	18	0	0	25	45	29	23	22	0	0	42
